# Delirium in Diabetic Ketoacidosis: A Case Report

**DOI:** 10.4274/Jcrpe.478

**Published:** 2012-03-08

**Authors:** Ayşe Nurcan Cebeci, Ayla Güven

**Affiliations:** 1 İstanbul Medeniyet University Göztepe Educational and Research Hospital, Pediatric Endocrinology, Istanbul, Turkey; +90 532 364 67 15nurcancebeci@yahoo.comgrowth

**Keywords:** Delirium, diabetic ketoacidosis, complication, brain edema, children Conflict of interest: None declared

## Abstract

A 15-year-old female patient with known type 1 diabetes mellitus was referred because of abdominal pain. On admission, she was alert but dehydrated with marked Kussmaul breathing. Blood glucose was 414 mg/dL (23 mmol/L). Blood gas analysis revealed severe metabolic acidosis (pH: 6.99) with an elevated anion gap (29.8 mmol/L) and an increased base excess (-25.2 mmol/L). At the sixth hour of treatment with intravenous fluids and insulin, the patient became delirious. The delirium persisted despite the normalization of the acidosis and became difficult to manage. Brain imaging studies revealed neither brain edema nor other intracranial pathology. No evidence of intoxication could be found. The patient gradually regained consciousness and was diagnosed as a case of severe diabetic ketoacidosis (DKA) associated with infection. We were unable to find a similar case in the pediatric literature and thought that reporting this unusual case would be a contribution to the literature on DKA in children.

**Conflict of interest:**None declared.

## INTRODUCTION

Neurological changes during the course of diabetic ketoacidosis (DKA) should be considered as early signs of cerebral edema and should be treated immediately. Delirium on the other hand, is not a usual neurological complication of DKA and has not been reported in pediatric DKA so far. We report here our experience with a teenage girl who developed hyperactive delirium during the treatment of DKA. 

## CASE REPORT

A 15 7/12 years old female patient was admitted with complaints of fatigue and abdominal pain. She was diagnosed as type 1 diabetes mellitus two years ago and had never experienced an attack of DKA at the time of diagnosis or at follow-up. In the past 24 hours, she had complained of abdominal pain, was not able to eat, and had poor appetite. Her glycemic control showed mild hypoglycemia (61 mg/dL, 3.4 mmol/L) in the morning of admission. Due to poor appetite and hypoglycemia, the patient omitted her insulin dose at lunch time; the same evening, she felt worse and was brought to our emergency department by her parents. On physical examination, she was alert, dehydrated, had deep sighing respirations and smell of ketones. Her height was 168 cm (+1.02 SDS), weight 68 kg (+1.66 SDS), body mass index 24.1 kg/m2 (+1.08 SDS); respiratory rate was 38/min, pulse 80/min. She had normal body temperature (36.4°C, axillary) and normal blood pressure (110/80 mmHg). Blood glucose was 414 mg/dL (23 mmol/L), capillary pH was 6.99 and bicarbonate 5.0 mmol/L. Base excess was -25.2 mmol/L and anion gap was 29.8 mmol/L. Blood urea, liver enzymes and electrolytes were within normal limits (Table 1). After the initial saline bolus of 400mL/m2 over the first hour, insulin infusion was started at a rate of 0.05 U/kg/h in an infusion fluid containing 2 parts saline:1 part 5% dextrose, with added potassium. 

At the 6^th^ hour of treatment, the patient became agitated and tried to get up and walk. She could not be appeased and attacked the staff. She ripped off her infusion sets three times in 30 minutes. With a suspicion of brain edema, her head was elevated, fluid infusion was restricted by 1/3 and mannitol infusion was started. A cerebral computed tomography was performed immediately and showed no brain edema or hemorrhage. She had high blood pressure (180/100 mmHg) but no bradycardia. In the mean time, the patient was very agitated and we had to strap her to the bed as she injured two nurses. The acidosis could not be corrected because the insulin and fluid therapy was interrupted. At the eighth hour, her Glasgow Coma Scale was 9. In consultation with an anesthesiologist, 1 mg of midazolam was given for sedation, thus, we were able to start an iv line. At the 10th hour, to correct the acidosis, bicarbonate infusion was initiated with insulin 0.1 u/kg/h though her bicarbonate level was 7 mmol/L. Mannitol infusion was stopped since there was no improvement in consciousness. At the 12^th^ hour, despite the improvement in acidosis, delirium still persisted. A brain magnetic resonance imaging (MRI) performed at this time was reported as normal. Substance abuse and drug intoxication were suspected. The patient’s blood pressure decreased to 130/90 mmHg, but tachypnea persisted. She was screaming, making incomprehensible sounds and was responding to painful stimuli. We continued with the same fluid infusion, but the rate of insulin was increased stepwise to 1.7 u/kg/h as serum glucose level did not decrease. At the 16th hour, the blood gas analyses showed a worsening (Table 2). This change was attributed to cessation of insulin and fluid infusion during the MRI. Therefore, the HCO3 infusion was repeated. 

At the 18^th^ hour of treatment, the patient developed a high fever. Serum C-reactive protein level was high (7.2 mg/dL; N: 0-0.8 mg/dL). Samples for blood and urine cultures were taken and iv ceftriaxone was started. Lumbar puncture was considered but postponed due to the instability of the patient. Retinal examination was normal. At this time, her agitated state disappeared and the patient began to sleep. At the 24th hour, acidosis had resolved completely, but she was still unconscious with little response to verbal stimuli. The patient had vulvovaginitis and treatment with fluconazole was started. In the following hours, consciousness improved slowly. At the 30^th^ hour of treatment, she could open her eyes in response to calling her name. Finally, at the 36^th^ hour, the patient was able to obey commands and sit up. The fluid and insulin infusions were stopped and subcutaneous insulin was started. The patient could not remember the delirium episode and no neurological sequelae were observed. She was discharged after a few days with complete recovery.

## DISCUSSION

We presented an unusual case with delirium due to severe acidosis. Correcting the acidosis alone was not enough to manage the delirium and the treatment was challenging. Delirium in patients treated in intensive care unit has been reported as a common and serious acute brain dysfunction with adverse outcome and high risk of mortality (1). Delirium is characterized by four features: 1) inattention and disturbance of consciousness, 2) change in cognition, 3) acute onset and fluctuating course, and 4) presence of a pathophysiological cause (2). Delirium can present as a hyperactive state, like in our patient, or a hypoactive state, which is often missed if not monitored closely (3). Once delirium is detected, the first aim is to identify the underlying etiology (4). Grover et al (5), in a study including 46 children and adolescents with delirium, reported that the most common underlying pathology was infection of various types, followed by neoplasms. The underlying pathology was severe acidosis associated with infection in our case. The delirium persisted in spite of the improvement of the acidosis; and we believe that the treatment of the infection had a major contribution to recovery. In pediatric delirium patients, both haloperidol and risperidone in low doses were found to be effective (6); however, since we have no experience with antipsychotic medications, we only used midazolam, which provided sedation for a short time period. The psychiatric evaluation of the patient after delirium was reported as normal. 

During the treatment of DKA, any change in the patient’s neurological state should be considered as a sign of cerebral edema (7). Our patient had signs of cerebral edema such as an abnormal respiratory pattern, altered mental state and high blood pressure. We started treatment for cerebral edema, but the patient did not respond to this therapy. Also, imaging studies revealed no findings consistent with cerebral edema or any other intracerebral causes of neurological deterioration such as thrombosis or hemorrhage. Severe hypocapnia and severe acidosis at presentation are potential risk factors for cerebral edema (8,9). Since our patient had no cerebral edema but did show these risk factors, we suggest that hypocapnia and acidosis are risk factors also for development of delirium in DKA. Although our patient was reported to have mild hypoglycemia in the morning of her presentation, which led her to omit her insulin dose, the hypoglycemia had improved by lunch time. Severe hypoglycemia is known to be associated with brain dysfunction, especially in young children (10,11), but the neurological symptoms are known to disappear after the plasma glucose concentration is raised (12,13). At admission, our patient was alert and had hyperglycemia, thus, we believe that the delirium developed as a result of severe acidosis rather than the episode of mild hypoglycemia.

Current guidelines (7) do not include delirium as a serious complication of DKA in children. In the literature, only a single case was reported with similar features (14). This was a 31-year-old female with known diabetes admitted with severe acidosis and delirium. Despite normalization of serum glucose and acidemia with treatment, the delirium persisted, as was also observed in our patient. This adult patient was also investigated for a toxic etiology, and the authors concluded that the patient “merely” suffered from DKA associated with cocaine use. We also suspected intoxication but did not analyze the serum. The patient’s parents appeared to be reliable individuals and they denied any substance abuse by their daughter. Thus, we accepted that our patient “merely” suffered from DKA associated with infection.

In summary, pediatricians should be aware that delirium can be seen in severe acidosis. Treatment of delirium can be challenging, especially when the staff has no experience in dealing with aggressive patients. Since there are not many similar cases in the literature, we wanted to share our experience regarding this 15-year-old female patient, hoping to contribute to knowledge on pediatric DKA.

## Figures and Tables

**Table 1:**
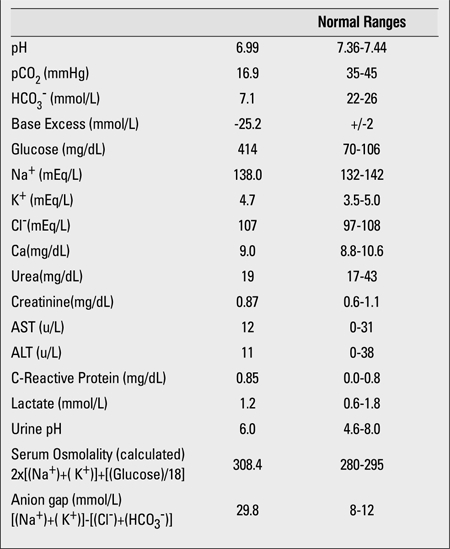
Laboratory values at admission

**Table 2:**
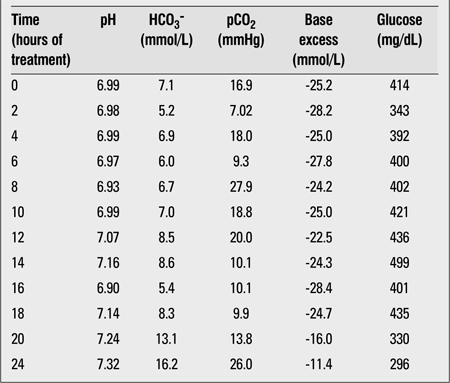
Capillary blood gas analyses and capillary glucosemeasurements of the patient during treatment of diabetic ketoacidosis
